# Thoracoscopy in Pleural Malignant Mesothelioma Diagnosis

**DOI:** 10.1155/DTE.3.147

**Published:** 1997

**Authors:** G. F. Tassi, G. P. Marchetti, F. Fattibene, P. L. Chiodera

**Affiliations:** 1 Division of Pneumology Valle Camonica Hospital Esine (Brescia) 25040 Italy; 2 Service of Pathologic Anatomy Valle Camonica Hospital Esine (Brescia) 25040 Italy

## Abstract

On the basis of our personal experience in 70 cases (66 pleural effusions) observed during the period January 1984- January 1996 we are here illustrating and discussing the diagnostic role of thoracoscopy in malignant pleural mesothelioma.

A histological diagnosis was achieved in 94.2% of cases. The endoscopic appearance was clearly neoplastic (masses, nodules) in 53 patients (75.7%) and simply inflammatory in 17 pachypleuritis in 13 (18.6%) and of diffuse hyperemia in 3 (5.7%). In all cases fluid cytology (diagnostic yield: 18.5%) and needle biopsy (diagnostic yield 17.1%) were performed.

The extension of pleural involvement (endoscopic staging according to Boutin) was also determined. In 16 patients (22.8%) a parietal and diaphragmatic involvement (stage Ia) was found. In 40 patients (57.2%) an associated visceral invasion (stage Ib). In 14 cases (20%) a diffuse parietal, visceral and mediastinal extension (stage II). The exam has always been well tolerated with few immediate complications: subcutaneous emphysema (4 cases) and some negligeable parietal bleeding (2 cases).


*Diagnostic and Therapeutic Endoscopy*, 1997, Vol. 3, pp. 147–151
Reprints available directly from the publisher
Photocopying permitted by license only

© 1997 OPA (Overseas Publishers Association)
Amsterdam B.V. Published in The Netherlands
by Harwood Academic Publishers
Printed in Singapore

### Thoracoscopy in Pleural Malignant Mesothelioma Diagnosis

G. F. TASSI, G. P. MARCHETTI, F. FATTIBENE and P. L. CHIODERA*

*Division of Pneumology and *Service of Pathologic Anatomy, Valle Camonica Hospital, 25040, Esine (Brescia), Italy*

*(Received 6 June 1996; revised 9 July 1996; in final form 7 August 1996)*

**On the basis of our personal experience in 70 cases (66 pleural effusions) observed during the period January 1984- January 1996 we are here illustrating and discussing the diagnostic role of thoracoscopy in malignant pleural mesothelioma.**

**A histological diagnosis was achieved in 94.2% of cases. The endoscopic appearance was clearly neoplastic (masses, nodules) in 53 patients (75.7%) and simply inflammatory in 17 pachypleuritis in 13 (18.6%) and of diffuse hyperemia in 3 (5.7%). In all cases fluid cytology (diagnostic yield: 18.5%) and needle biopsy (diagnostic yield 17.1%) were performed.**

**The extension of pleural involvement (endoscopic staging according to Boutin) was also determined. In 16 patients (22.8%) a parietal and diaphragmatic involvement (stage Ia) was found. In 40 patients (57.2%) an associated visceral invasion (stage Ib). In 14 cases (20%) a diffuse parietal, visceral and mediastinal extension (stage II).**

**The exam has always been well tolerated with few immediate complications: subcutaneous emphysema (4 cases) and some negligeable parietal bleeding (2 cases).**

*Keywords:* pleural mesothelioma, diagnosis, thoracoscopy, pleural effusion

### INTRODUCTION

There are still considerable difficulties in the diagnosis of pleural mesothelioma, both because of its aspecific clinical manifestations and because of the limited sensitivity of traditional X-rays and of computed axial tomography.

Fluid cytology and needle biopsy have only partially improved chances of identifying the pathology (Whitaker and Shikin 1984; Leong *et al.* 1992).

Thoracoscopy however, which has become a much more widely used method in recent years, in expert hands allows a correct diagnosis to be made in nearly all cases, thereby equalling the accuracy previously produced only by thoracot-

Address of the corresponding author: Gianfranco Tassi MD, Divisione di Pneumologia, Ospedale di Valle Camonica, 25040 Esine (Brescia), Italy.

omy (Boutin and Rey 1993). The procedure not only allows adequate and abundant tissue sampling for immunohistochemical staining which is essential for differential diagnosis from adenocarcinoma (Leong and Vernon-Roberts 1994) but also allows staging of the disease which is a useful prognostic factor and an important element in deciding therapy (Boutin *et al.* 1993).

This report aims at illustrating and discussing the diagnostic role of thoracoscopy in mesothelioma on the basis of our experience in 70 cases.

### MATERIALS AND METHODS

70 cases of pleural malignant mesothelioma were diagnosed at thoracoscopy first in the Pneumology Division of the Spedali Civili in Brescia (1984–1986) and then in the Pneumology Division of the Ospedale di Valle Camonica (1987–1996) (Table I), between January 1984 and January 1996. During this period for diagnostic purposes in pleural disease of unknown etiologyb we carried out a total of 470 thoracoscopies so the disease was diagnosed in 14.9% of the diagnostic examinations.

49 of the patients were male (70%) and 21 were female (30%) with a M/F ratio of 2.3/1. Indications for thoracoscopy were pleural effusion in 66 patients (94.2%), while the existence of pleural thickening without exudation was the only anomaly found in 4 (5.8%). The right side was affected in 36 cases (51.4%), the left in 34 (48.6%), there were no bilateral cases. The average age was 61 years (min 41 yrs, max 84 yrs). Anamnesis for occupational exposure to asbestos was definitely positive in only 17 (24.2%) cases and negative or not clear in the remaining 53.

TABLE I Thoracoscopy in Pleural Mesothelioma

| | Personal experience (1984-1996) | |
|---|---|---|
| Patients (n) | 70 | (M49; F21) |
| Patients aged < 60 a. (n) | 28 | (40%) |
| Average age (yrs) | 61.4 | |
| Pleural effusion (n) | 66 | (94.2%) |

All patients underwent pleural fluid cytology and closed pleural biopsy by Cope needle. The day before thoracoscopy, any pleural fluid present was aspirated and pneumothorax was then induced using a Morelli device; intrapleural pressures were determined which together with radioscopic control checked the degree of parenchymal collapse, according to our usual technique (Tassi and Marchetti 1993). The average amount of air insufflated was 100–300 ml, which gave a space of at least 5 cm between the lung and the chest wall.

The actual thoracoscopy was always performed under local anesthesia (10–15 ml idocaine 2%), inserting Boutin's rigid thoracoscope (Wolf Co., Knittlingen, Germany) connected to a video camera for monitor viewing into the 5th -6th intercostal space on the midaxillary line using a single port. A second point of entry was necessary only in 4 cases in order to reach areas otherwise inaccessible. The entire pleural area was always explored and multiple biopsy samples (5–10) were taken both in any suspicious areas and in sites apparently without macroscopically evident lesions. In 3 patients with apparently normal visceral pleura and coexistent fibrohyaline parietal plaques, pleural biopsies were integrated with samples from the lung parenchyma using a coagulating forceps.

At the end of the endoscopy, when indicated, poudrage was performed using an asbestos-free talc to induce symphysis (2.5–5 g): 29 patients were so treated.

In all cases a chest tube for drainage (24–28 Fr) was inserted and left in place for an average of 3 days (min. a few hours, max 7 days). Since 1992, 39 patients have undergone local preventive radiotherapy 10–15 days after the examination, with a total dose of 21 Gy over 3 days to a depth of 3 cm over an area between 50 and 100 $cm^2$ centered over each point of entry (Boutin *et al.* 1995).

### RESULTS

Exploration of the pleural cavity was straight forward in 59 patients (84–2%) since in these cases there were no adhesions, whereas in 11 patients (15.8%) complete exploration was made difficult; by the presence of adhesions which, even after lysis with forceps, sometimes partly hindered the procedure. However even in these cases the biopsies taken usually allowed a correct diagnosis to be made.

Most of the lesions observed (Table II) were clearly neoplastic (Fig. 1): isolated nodules or multiple lesions (vegetations; masses; thickenings). Diffuse pachypleuritis was found less frequently (Fig. 2) and simple inflammation (Fig. 3) only rarely. Moreover the presence of fibrohyaline plaques with the characteristics of asbestotic plaques was noted in 13 patients (18.6%).

Biopsy allowed the diagnosis of pleural malignant mesothelioma in 66 patients (94.2%) and also provided its histological classification (Table III) with a marked prevalence of epithelial type. In 2 cases in which biopsies were insufficient due to extensive adhesions which prevented a complete exploration of the cavity diagnosis was made after thoracotomy. In another 2 cases diagnosis was reached with a second thoracoscopy performed some months later due to the reappearance of the effusion. In all 3 patients submitted to forceps parenchymal biopsy we obtained a diagnosis of pulmonary asbestosis without complications using a coagulating forceps which insured the aerostasis of the lung therefore avoiding air leak.

TABLE II Pleural Mesothelioma (n 70)

| | Endoscopic pictures |
|---|---|
| | % |
| Inflammation | 4 (5.7) |
| Nodules | 17 (24.3) |
| Pachypleuritis | 13 (18.6) |
| Multiple lesions | 36 (51.4) |

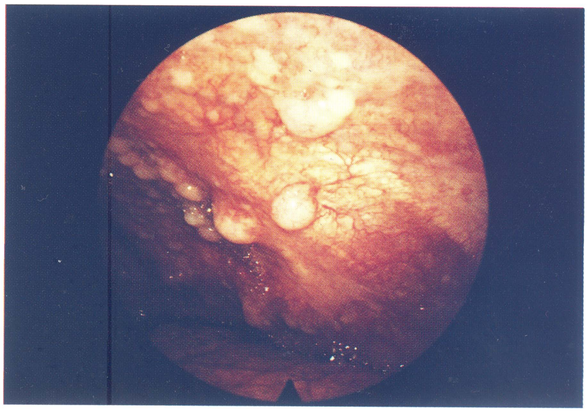

FIGURE 1

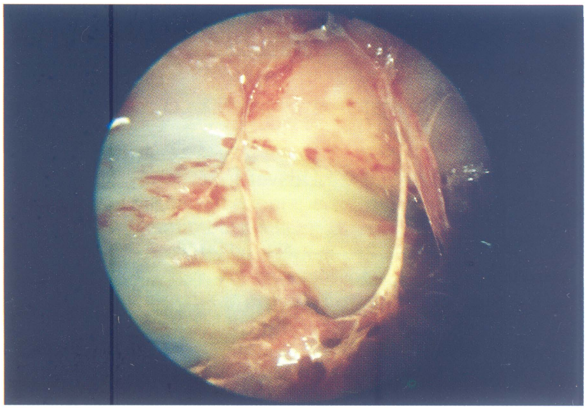

FIGURE 2

Thoracoscopy also allowed Boutin classification of the disease according to the degree of pleural involvement: in 16 cases (21.8%) only the parietal and diaphragmatic pleura were involved (stage Ia), 40 (57.2%) presented visceral invasion (stage Ib), and 14 (20%) massive invasion of all the pleural surfaces (stage II).

Talc poudrage made in 29 cases achieved a permanent pleurodesis with no recurrence of effusion in 24 patients (83%).

Thoracoscopy was always well tolerated and there were no major complications immediately after the procedure. Minor complications included subcutaneous emphysema in 4 patients (5.7%) and minor parietal bleeding in 2 (2.8%). Later complications included 3 (4.3%) cases of

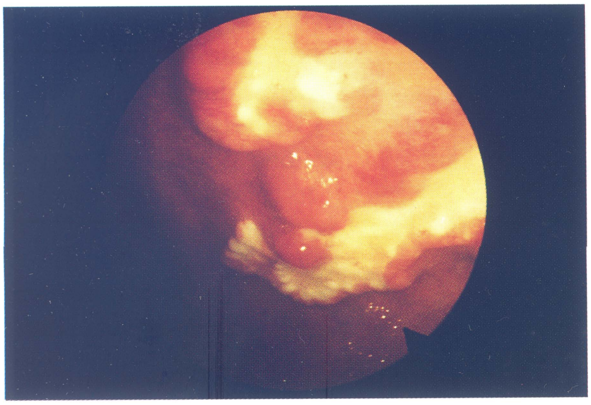

FIGURE 3

TABLE III Pleural Mesothelioma (n 70)

| | Histological type |
|---|---|
| | % |
| Epithelial | 55 (78.6) |
| Sarcomatous | 5 (7.1) |
| Mixed cell | 7 (10) |
| Other | 3 (4.3) |

neoplastic invasion of the thoracic scar, all of them in the first 31 patients not treated by local radiotherapy. This no longer occurred in the remaing 39 who had a radiation therapy.

### DISCUSSION

In recent years thoracoscopy has been enjoying a second youth and has become an invaluable method in the diagnosis of pleural effusions in general and mesothelioma in particular. In expert hands the examination is diagnostic in the vast majority of cases, reducing the need for exploratory thoracotomy which has higher morbidity and mortality (Weatherford *et al.* 1995).

If we compare data regarding the sensitivity of the methods used before thoracoscopy the clear advantage of the latter is striking. Our experience (Table IV), similar in results to other large case studies, shows that mesothelioma can be diagnosed by thoracoscopy in 94% of cases: much higher than the 17/18% diagnostic yield of needle biopsy and fluid cytology.

TABLE IV Pleural Mesothelioma (n 70)

| | Diagnostic means |
|---|---|
| | % |
| Fluid cytology | 13/70 (18.5) |
| Pleural needle biopsy | 12/70 (17.1) |
| Thoracoscopic biopsy | 66/70 (94.2) |

Even thoracoscopy does not however reach 100% sensitivity. The small percentage of false negatives present in all the studies is usually due to the presence of adhesions which cannot be divided endoscopically and which therefore prevent a complete exploration or the pleural cavity. It should be noted that this is usually the case when thoracoscopy is delayed too long and often eventually performed several months after the appearance of an effusion. It can therefore be supposed, and our experience confirms this, that a prompt use of the method in the investigation of pleurisies which have not been diagnosed with other means can further increase sensitivity.

Clearly neoplastic lesions (vegetations; nodules; multiple lesions) were present in 75.7% of our cases showing that a generic diagnosis of malignancy at endoscopy is possible in most patients. However there can be more deceptive pictures of simple inflammation or of generic pachypleuritis (1/4 of our patients) and these cases emphasize the need for multiple biopsies from a wide range of sites.

Asbestotic type fibrohyaline plaques (an irregular surface with a 'candle wax drop' appearance), which we observed in 13 (18.6%) of our 70 patients are also useful for diagnosis: these plaques found together with clearly neoplastic lesions (nodules or masses) (Fig. 4) clearly point to a diagnosis of mesothelioma. In fact during our experience in neoplastic pleurisy in 400 thoracoscopies we have never found these plaques associated with other tumors. Another useful point to remember is that when fibrohyaline

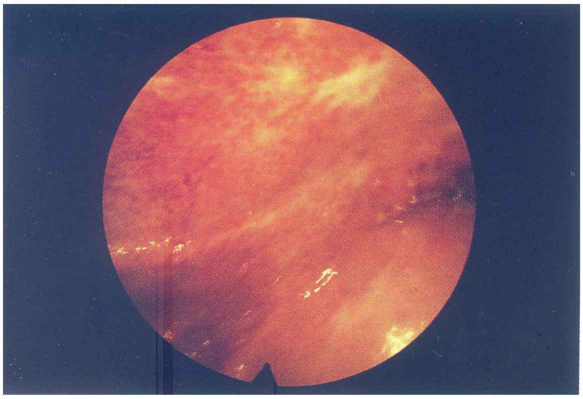

FIGURE 4

plaques are found but occupational exposure has not been proved, lung biopsy with coagulating forceps can be performed during the same thoracoscopy session and under local anesthesia, thereby demonstrating an eventual asbestosis.

We, like other authors (Rusch 1995), used thoracoscopic staging for therapeutic approach. In fact only fairly recently has it been discovered that mesothelioma which has invaded the visceral pleura after primitive involvement of the parietal and diaphragmatic pleura has a much worse prognosis and that in its early stages the disease may respond to an efficacious treatment (Astoul *et al.* 1993, Boutin *et al.* 1994).

Therefore, latterly, 3 stage Ia patients were treated surgically with pleurectomy and 6 with intracavitary drugs (immunotherapy with gamma-interferon and chemotherapy with cisplatin) after the positioning of a device for intrapleural infusion. The number of patients treated is too small to be able to draw conclusions but we consider the clinical results of this approach to be encouraging. Obviously this treatment was possible only after thoracoscopy, once again underlining its usefulness in the modern clinical/therapeutical management of pleural tumor.

In the case of more extensive involvement (stages Ib and II) in patients with massive and recurrent effusions we performed talc poudrage which is still generally considered as the best technique of pleurodesis (Walker-Renard *et al.* 1994). Its complete efficacy in 83% of the cases avoided the need for painful thoracentesis which also exposes patients to the risk of neoplastic seeding.

Tolerance to the examination was very good, and there were no fatalities which further proves that thoracoscopy, if correctly performed, presents no particular risks or complications.

We can therefore conclude that the role of thoracoscopy in mesothelioma nowadays is undeniably fundamental, both for diagnostic and therapeutic purposes, and it should be systematically utilized in all suspicious cases.

### *References*

[1] Whitaker, D., Shilkin, K. B.: Diagnosis of pleural mesothelioma in life: a practical approach. *J. Pathol.*, 1984; **143**: 147–175.

[2] Leong, A. S. Y., Stevens, C. T., Mukherjee, T. M.: Malignant mesothelioma: cytologic diagnosis with histologic, immunohistochemical and ultrastructural correlation. *Semin. Diag. Pathol.*, 1992; **9**: 141–150.

[3] Boutin, C., Rey, F.: Thoracoscopy in pleural malignant mesothelioma: a prospective study of 188 consecutive patients. Part 1: Diagnosis. *Cancer*, 1993; **72**: 389–393.

[4] Leong, A. S .Y., Vernon-Roberts, E.: The immunohistochemistry of malignant mesothelioma. In: Rosen P. P. and Fechner R. E. (eds.): *Pathology Annual*, Part 2. Appleton & Lange, Norwalk, 1994.

[5] Boutin, C., Rey, F., Gouvernet, J. *et al.*: Thoracoscopy in pleural malignant mesothelioma: a prospective study of 188 consecutive patients. Part 2: Prognosis and staging. *Cancer*, 1993; **72**: 394–404.

[6] Tassi, G. F., Marchetti, G. P.: *Toracoscopia*. Acram, Milano, 1993.

[7] Boutin, C., Rey, F., Viallat, J. R.: Prevention of malignant seeding after invasive diagnostic procedure in patients with pleural mesothelioma: a randomized trial of local radiotherapy. *Chest*, 1995; **108**: 754–758.

[8] Weatherford, D. A., Stephenson, J. E., Taylor, S. M. et al.: Thoracoscopy versus thoracotomy: indications and advantages. *Am. Surg.*, 1995; **61**: 83–86.

[9] Rusch, V. W.: A proposed new international TNM staging system for malignant pleural mesathelioma. *Chest*, 1995; **108**: 1122–1128.

[10] Astoul, P., Viallat, J. R., Laurent, J. C. et al.: Intrapleural recombinant IL-2 passive immunotherapy for malignant pleural effusion. *Chest*, 1993; **103**: 209–213.

[11] Boutin, C., Nussbaum, E., Monnet, I. *et al.*: Intrapleural treatment with recombinant gamma interferon in diffuse malignant mesothelioma. *Cancer*, 1994; **74**: 2460–2467.

[12] Walker-Renard, P., Vaughan, L. M., Sahh, S. A.: Chemical pleurodesis for malignant pleural effusions. *Ann. Intern. Med.*, 1994; **120**: 56–64.

